# Mesenchymal Stem Cell-Derived Exosomes Modulate Chondrocyte Glutamine Metabolism to Alleviate Osteoarthritis Progression

**DOI:** 10.1155/2021/2979124

**Published:** 2021-12-27

**Authors:** Kai Jiang, Ting Jiang, Yang Chen, Xinzhan Mao

**Affiliations:** ^1^Department of Orthopedics, The Second Xiangya Hospital, Central South University, China; ^2^Department of Orthopedics, The Third Xiangya Hospital, Central South University, China

## Abstract

Osteoarthritis (OA) had a high incidence in people over 65 years old, and there is currently no drug that could completely cure it. This study is aimed at studying the role of exosomes in regulating glutamine metabolism in the treatment of OA. First, we identified the exosomes extracted from the mouse OA model's bone marrow mesenchymal stem cells (MSC). *In vitro*, compared with the control group, the cell apoptosis in the OA group increased, while the cell proliferation of the OA group was suppressed. After exosomal treatment, cell apoptosis and cell proliferation were reversed. Inflammatory factors (TNF*α*, IL-6), glutamine metabolic activity-related proteins (c-MYC, GLS1), glutamine, and GSH/GSSG were increased in the OA group. The overexpression of c-MYC reduced the therapeutic effect of exosomes. At the same time, we found that chondrocyte functional factors (collagen II, Aggrecan) were improved under the treatment of exosomes. However, oe-c-MYC reversed the therapeutic effect of exosomes. *In vivo*, we found that the running capacity of the mice in the OA group was reduced, and the cartilage tissue was severely damaged. In addition, TNF*α*, IL-6, and chondrocyte apoptosis increased, while the metabolism of collagen II, Aggrecan, and glutamate decreased in the OA group. After exosomal treatment, the mice's exercise capacity, tissue damage, inflammation, and chondrocyte function were improved, and glutamate metabolism was increased. This study showed that exosomes regulated the level of chondrocyte glutamine metabolism by regulating c-MYC, thereby alleviating OA.

## 1. Introduction

Osteoarthritis (OA) is a common delayed and degenerative disease. Among the elderly over 60 years old, about 10% of men and 18% of women are deeply troubled by it [1]. Age, obesity, metabolic syndrome, genetic susceptibility, disorganization, acute joint injury, and decreased sex hormone levels may be risk factors for OA [2, 3]. Pain is the biggest symptom of OA patients. In addition, OA can also cause joint swelling and spasm, which limit people's daily activities [3]. So far, there is no way to cure OA completely. People can only relieve pain and stiffness by changing risk factors, such as physical therapy (PT) and pharmaceutical treatment [3]. For example, previous studies have shown that weight loss can effectively reduce the knee load of OA patients to improve OA symptoms [4]. Another study showed that supervised exercise, personalized manual therapy, and a family exercise program could effectively reduce the OA index and relieve OA symptoms [5]. Some drugs such as glucosamine and chondroitin, acetaminophen, and celecoxib can be used to reduce the pain of OA [6]. However, these treatments can only relieve pain and control symptoms but cannot cure OA. Cartilage cells and inflammation are considered to play an important role in OA, but the current research on the pathogenesis of OA is still not thorough enough. Therefore, we urgently need to study the pathogenesis and treatment of OA.

In recent years, with the increasing research on exosomes, we found that exosomes have extensive and unique advantages in disease diagnosis and treatment [7]. Due to the endogeneity of exosomes, it can prevent the body from producing antibodies to exosomes and make exosomes have better biocompatibility [7]. And because of the heterogeneity of exosomes, they can easily carry various proteins on the surface and then enter cells [8]. Exosomes secreted by mesenchymal stem cells (MSC) have a significant role in the treatment of skin restenosis [9], myocardial ischemia/reperfusion injury [10], and inflammatory diseases [11].

Glutamine could be hydrolyzed to ammonium ion (NH4^+^) and glutamic acid, and then, glutamic acid was converted to glutamic acid *α*-ketopentanate (*α*-KG) [12]. After that, *α*-KG entered the tricarboxylic acid cycle (TCA cycle) and generated adenosine triphosphate (ATP) by producing nicotinamide adenine dinucleotide (NADH) and flavin adenine dinucleotide (FADH_2_) [13]. ATP is an essential fuel for cell activity. Therefore, the higher the content of glutamine, the more conducive to cell activity [13]. Reduced glutathione (GSH) is a metabolite of glutamine [14]. Glutathione depletion results in exacerbation of damage by oxidative and nitrosative stress and increased levels of proinflammatory mediators [15]. The ratio of reduced glutathione to oxidized glutathione (GSH/GSSG) determines the activation of c-Jun n-terminal kinase (JNK) and mitogen-activated protein kinase (MAPK) pathways, thereby determining proinflammatory cell transcription of factor [15]. Moreover, GSH is an antioxidant in the cell, and GSH/GSSG can be used as a marker of cell antioxidant capacity [16]. The transcription of factor c-MYC was widely involved in the key processes of normal cell proliferation, differentiation, and metabolism [17]. Studies have shown that c-MYC can enhance glutamate metabolism by increasing glutaminase (GLS) [17]. But in OA, whether c-MYC could regulate glutamate metabolism was not yet known. Synovial fluid (SF) is located in the articular cavity, which has a lubrication function and contains protein/metabolite markers of systemic diseases [18]. A previous study has shown that the content of glutamine in OA synovial fluid (SF) was higher than that of rheumatoid arthritis (RA), indicating that there were different expressions in glutamine metabolism [18]. Glutamine has an antioxidant function in OA and other inflammatory diseases [2]. Therefore, based on the above research, we intend to explore the role of MSC-exosomes in regulating glutamine metabolism of chondrocytes in OA and provide a new treatment for OA.

## 2. Materials and Methods

### 2.1. Animal Experiments

Thirty male Sprague Dawley (SD) rats were purchased from Hunan Sja Laboratory Animal Co., Ltd. The rats were adaptively fed for one week. The rats were randomly divided into three groups: sham group, OA group, and OA+exosome group, with 10 rats in each group. Rats in the sham group were only treated with incision and suture of the joint capsule. The OA model was established by anterior cruciate ligament transection (ACLT) [19]. Ketamine/xylazine was injected intraperitoneally according to body weight. The corneal reflex and toe reflex were observed. After the disappearance, the skin of both hind limbs was prepared, iodophor alcohol disinfection was carried out, and a sterile operation sheet was laid. The skin, fascia, and tendon were cut along the left and right knee joints' inner edge of the patellar tendon to dislocate the patella laterally. The anterior cruciate ligament was cut under direct vision. After the operation, the incision was sutured layer by layer and the skin around the incision was disinfected with alcohol. Four weeks later, cartilage wear was determined. The OA+exosome group rats were used to establish the ACLT model of bilateral hind limb joint injury. Four weeks later, after cartilage wear was determined; 100 *μ*g of exosomes in 50 *μ*l of PBS was injected into the joint cavity of the bilateral hind. After euthanasia, the tissue and peripheral blood were collected.

### 2.2. Preparation of Exosomes

According to the method of 2.1, the OA rat model was established, and MSCs were isolated from bone marrow. The MSCs were cultured in the DMEM medium containing 10% fetal bovine serum (FBS; GIBCO) and 1% penicillin-streptomycin. When the cell density was about 80%, the MSCs were digested by trypsin and passaged in two. The MSCs were cultured, and exosomes were prepared. After cells were cultured in a serum-free medium for 24 h, the cell supernatant was collected after centrifuging at 3,000 rpm for 15 min to remove cells and debris. The exosomes were precipitated from the supernatant by centrifugation at 100,000 rpm for 1.5 h at 4°C. The culture supernatant was removed. The sediment at the bottom was the exosomes. The exosomes were carefully immobilized with 1 ml of 2.5% glutaraldehyde for 1 h.

### 2.3. Cell Culture

Chondrocytes were isolated from rat knee joints. The cells were divided into three groups: control group, OA group, and OA+exosome group. The cells were seeded on a 6-well plate with 3 multiple wells in each group. Control group cells were cultured normally. OA group cells were treated with 10 ng/ml IL-1*β* [20]; in the OA+exosome group, the OA model was established and the cells were treated with 5 *μ*g/ml exosomes for 48 h. In addition, to study whether MSC-derived exosome regulated chondrocyte glutamate metabolism through c-MYC, we established a c-MYC overexpression cell line of rat chondrocyte. The c-MYC overexpression plasmid oe-c-MYC and the overexpression empty plasmid oe-NC were constructed in HonorGene Co., Ltd., and they were used to treat the cells of the OA+exosome group to detect the regulatory effect of c-MYC on the cells. The cells were divided into five groups: control group, OA group, OA+exosome group, OA+exosome+oe-NC group, and OA+exosome+oe-c-MYC group. The control, OA, and OA+exosome groups were treated with the same way as before. The OA+exosome+oe-NC and OA+exosome+oe-c-MYC groups were transfected with oe-NC and oe-c-MYC, respectively.

### 2.4. Running Tests

After 4 weeks of modeling, 5 rats in each group were kept and fed until the sixth week. All rats were placed on the treadmill. When the rate was set at 20 m/min, rats in the OA and sham groups did not move. When the rate was set at 15 m/min, the rats in the OA group did not move under 1 mA electrical stimulation while the sham group moved. The treadmill speed was set at 10 m/min, and all rats moved. When the rats stopped moving, we stimulated them with 1 mA current. All groups of rats were moving 37.69 m. Finally, we counted the number of shocks.

### 2.5. Hematoxylin-Eosin (HE) Staining

After the knee joint was fixed for 24 h, it was embedded in paraffin. The embedded tissue was sectioned. After baking at 60°C for 12 h, the slides were dewaxed to water. After dyeing with hematoxylin and eosin, gradient alcohol (95-100%) was dehydrated for 5 min. The slides were removed and placed in xylene for 10 min, twice. After sealing with neutral gum, the specimens were observed under a microscope and photographed.

### 2.6. Safranin Solid Green Staining

Similarly, the tissue sections were dewaxed to water. After the slides were stained with solid green dye solution, they were stained with safranin and washed with distilled water. A hairdryer was used to dry the slides. The slides were placed in xylene for 10 min, twice. Neutral gum was used to mount the sections and observe the slides with a microscope and took photos.

### 2.7. Terminal Deoxynucleotidyl Transferase-Mediated dUTP-Biotin Nick End Labeling (TUNEL) Assay

Similarly, the tissue sections were dewaxed to water. The slides were stained according to the TUNEL Kit (Shanghai Yisheng biology, No. 40306es50). Each slide was dripped with 100 *μ*l of Proteinase K working solution and reacted at 37°C for 20 min. The slides were rinsed with PBS. 100 *μ*l 1 × Equilibration Buffer was added to each slide, and the slides were incubated at room temperature for 10 min. Most of the 1 × Equilibration Buffer was absorbed with an absorbent paper. Then, 50 *μ*l of TdT buffer was added to the slides. The slides were incubated at room temperature for 60 min, and then they were rinsed with PBS. After the slides were stained, DAPI working solution was used to stain the nucleus for 10 min. After mounting the slides, it was observed and pictures were taken under a microscope. The number of apoptotic cells was counted.

### 2.8. Immunohistochemical (IHC) Assay

Similarly, the tissue sections were dewaxed to water. According to the manufacturer's instructions, the slides were stained with a two-step Kit (ZSGB-BIO, No. pv-8000). An appropriate amount of endogenous peroxidase blocker was added to the slides. And the slides were incubated for 10 min at room temperature. The slides were rinsed with PBS. The slides were dropped with 100 *μ*l primary antibodies (c-MYC, GLS) and incubated at 37°C for 60 min. PBS was used to rinse the sections. 100 *μ*l of secondary antibodies (anti-rabbit, rabbit, rabbit-IgG antibody-HRP polymer) was added dropwise. And the slides were incubated at room temperature for 20 min. The sections were rinsed with PBS. An appropriate amount of freshly prepared DAB chromogenic solution was added to the slides. Hematoxylin was used to restain the slides for 5-10 min, and the slides were rinsed with distilled water. PBS was used to turn the slides blue. The slices were dehydrated, cleared, and mounted. At last, the slides were observed under a microscope and photos were taken.

### 2.9. Real-Time Quantitative PCR (RT-qPCR)

Trizol kit was used to extract total RNA from tissues and cells. The concentrations of RNA were 100 ng/*μ*l-200 ng/*μ*l, and OD260/OD280 was 1.8-2.0. The HiFscript cDNA synthesis kit was used to reverse the mRNAs into cDNAs according to the manufacturer's instructions. *β*-Actin was used as an internal control. The reaction was predenatured at 95°C for 10 min. Then, the reaction was denatured at 95°C for 15 s, annealed and extended at 60°C for 30 s and subjected to 40 denaturation cycles. Primer sequences were used as follows: *β*-actin—F: ACATCCGTAAAGACCTCTATGCC, *β*-actin—R: TACTCCTGCTTGCTGATCCAC; TNF*α*—F: CCCCTCTATTTATAATTGCACCT; TNF*α*—R: CTGGTAGTTTAGCTCCGTTT; IL-6—F: TCACTATGAGGTCTACTCGG; IL-6—R: CATATTGCCAGTTCTTCGTA; Aggrecan—F: ACAGACACCCCTACCCTTGC, Aggrecan—R: CCTCACATTGCTCCTGGTCGAT; c-MYC—F: ACTCGGTGCAGCCCTATTTC, c-MYC—R: GTAGCGACCGCAACATAGGA; and GLS1—F: CTGCTGCAGAGGGTGGAATA, GLS1—R: GAGGTGTGTACTGGACTTGGT.

### 2.10. Western Blot (WB)

According to the instructions of the BCA protein quantification kit, extract the protein of exosomes in the cell or cell supernatant, and determine the protein concentration. The protein was separated by 10% SDS-PAGE gel and then transferred to the PVDF membrane (Millipore, Bedford, MA, USA). 5% skimmed milk powder was prepared with 1 × PBST, and the membrane was immersed and placed at room temperature for 90 min. The primary antibodies TNF*α* (1 : 1,000, No. ab6671, Abcam), IL-6 (1 : 1,000, rabbit, bs-0782r, bioss), c-MYC (1 : 10,000, rabbit, ab32072, Abcam), GLS1 (1 : 1,000, rabbit, ab156876, Abcam), CD63 (1 : 1,000, rabbit, ab134045, Abcam), CD9 (1 : 1,000, rabbit, ab92726, Abcam), CD81 (1 : 1,000, rabbit, ab109201, Abcam), GM130 (1 : 1,000, rabbit, ab52649, Abcam), and *β*-actin (1 : 5,000, mouse, 66009-1-Ig, Protein) were diluted in proportion with 1 × PBST; *β*-actin was an internal control. The membrane was incubated with the primary antibody for 90 min at room temperature. The secondary antibodies HRP goat anti-mouse IgG (1 : 5,000, SA00001-1, Proteintech) and HRP goat anti-rabbit IgG (1 : 6,000, SA00001-2, Proteintech) were diluted with 1 × PBST. The diluted secondary antibody was incubated with the membrane for 90 min at room temperature. ECL was used to develop color exposure. Finally, grayscale analysis was performed.

### 2.11. Enzyme-Linked Immunosorbent Assay (ELISA)

The peripheral plasma of rats in each group was collected. According to the manufacturer's instructions, the ELISA kit (Enzyme Linked Bioengineering Co., Ltd., No. ml059460) was used to detect *α*-KG. The cell supernatant of each group was collected. The biochemical kits were used to detect GLN (Nanjing Jiancheng Institute of Biology, No. A073-1-1), GSH (Nanjing Jiancheng Institute of Biology, No. A006-2-1), and GSSG (Nanjing Jiancheng Institute of Biology, No. A061-1-1).

### 2.12. The Cells Were Identified by Flow Cytometry

The cells were cultured in 6-well plates and treated according to the groups. After reaching the predetermined time, the cells were digested. The cells were labeled with CD29, CD106, CD34, and CD45 antibodies, respectively. In MSC, CD29, CD106, and CD34 were positive, while CD34 and CD45 were negative.

### 2.13. Transmission Electron Microscopy

PBS was used to resuspended exosomes. Approximately 10 *μ*g of exosomes resuspended in PBS was dropped on the para membrane. The exosomes were examined by an electron microscope and photographed.

### 2.14. Cell Apoptosis Assay

The cells were cultured in 6-well plates and treated according to the groups. After reaching the predetermined time, the cells were digested. After the cells were suspended in binding buffer, 5 *μ*l V-FITC and 5 *μ*l PI were mixed with the cells. After 10 min of dark reaction, mixtures were detected by the flow cytometer.

### 2.15. Statistical Analysis

All experiments were repeated 3 times, and the data were expressed as the average value ± standard deviation (SD). GraphPad Prism 9.0 was used to analyze the data. For the comparison between the two groups, we used Student's *t*-test to compare. For more than two independent groups, we used one-way ANOVA to compare. And the Tukey test was added if necessary. *P* < 0.05 was considered statistically significant.

## 3. Results

### 3.1. Isolation of Bone Marrow MSCs and Uptake of Exosomes

**Figure 1:**
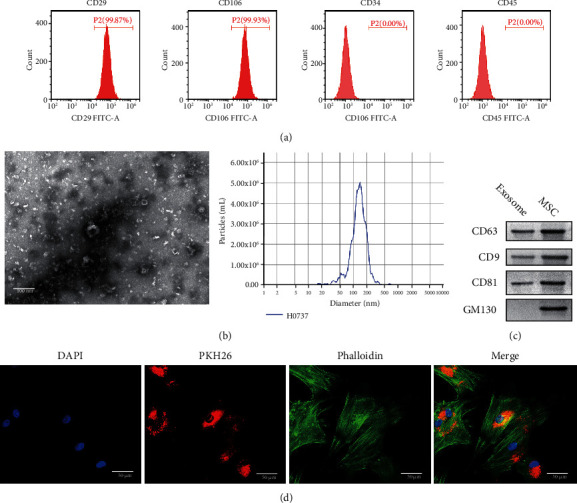
Isolation of bone marrow MSCs and uptake of exosomes. (a) The contents of CD29, CD106, CD34, and CD45 in exosomes were detected by flow cytometry. (b) The diameter of exosomes was detected by an electron microscope. Scale bar = 100 nm. (c) The exosome-associated proteins CD63, CD9, and CD81 were detected by WB. (d) IF was used to detect the uptake of exosomes by chondrocytes. DAPI was blue fluorescence, PKH26 was red fluorescence, phalloidin and was green fluorescence. Scale bar = 50 *μ*m.

To determine the therapeutic effect of MSC secreted exosomes on OA, we first established an OA rat model and extracted MSC from the spinal cord. To identify whether the cells were MSC, we used flow cytometry to identify the cells. The results showed that CD29 and CD106 were positive and CD34 and CD45 were negative, which indicated that the cells were indeed MSC (Figure 1(a)) [21]. MSCs were cultured and exosomes were isolated from a serum-free supernatant. The exosomes were identified by electron microscopy (Figure 1(b)). The size of exosomes is generally 40-100 nm [22]. Then, we detected the contents of exosomal marker proteins CD63, CD9, CD81, and GM130 in exosomes and MSC by WB [23]. The results showed that CD63, CD9, and CD81 were expressed in exosomes and MSC, while the GM130 protein was only expressed in MSC (Figure 1(c)). Therefore, the substance we extracted is indeed exosomes. PKH67 is a widely used exosomal marker [24, 25]. Exosomes were labeled with PKH67; cytoskeletons were labeled with F-actin and phalloidin combined with F-actin to prevent its depolymerization and poisoning cells. Fluorescence staining showed that the exosomes could be absorbed by chondrocytes (Figure 1(d)).

### 3.2. Exosomes Affect Cell Function and Glutamine Metabolism of OA Chondrocytes

**Figure 2:**
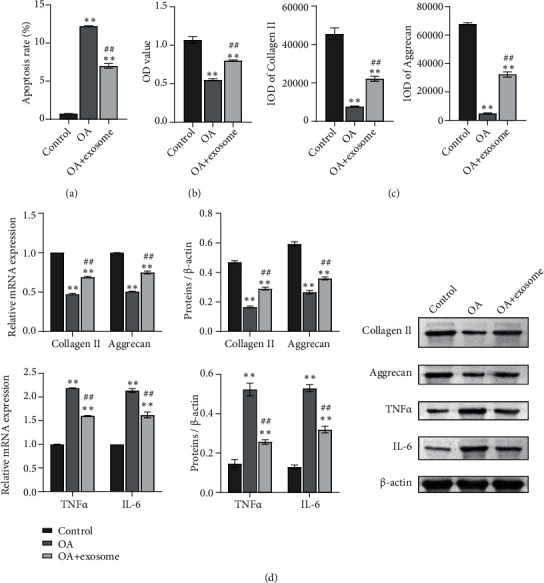
Exosomes affect cell function of OA chondrocytes. (a) Apoptosis was identified by flow cytometry. (b) The CCK8 method was used to detect cell proliferation. (c) Collagen II and Aggrecan of chondrocytes were detected by IF. (d) The mRNA and protein contents of collagen II, Aggrecan, TNF*α*, and IL-6 were detected by RT-qPCR and WB. ^∗∗^*P* < 0.01 vs. control; ^##^*P* < 0.01 vs. OA.

To determine whether there are differences in the cell function and glutamate metabolism between the control, OA, and OA+exosome groups, we established the OA rat chondrocyte model *in vitro*. Cell apoptosis rate was identified by flow cytometry. The results showed that the apoptosis rate of the OA group was significantly higher than that of the control group, and exosome treatment reversed the increase of apoptosis (Figure 2(a), Figure S1A). Then, we used the CCK8 assay to detect the proliferation of cells. We found that compared with the control group, the proliferation rate of the OA group decreased, and exosome treatment reversed the effect of OA (Figure 2(b)). Collagen II and Aggrecan of chondrocytes were detected by IF to detect the function of chondrocytes. The results showed that OA decreased the expression of collagen II and Aggrecan, while exosomes played a positive role (Figure 2(c), Figure S1B). Furthermore, we detected the contents of collagen II and Aggrecan by RT-qPCR and WB, and the results were consistent with the results (Figure 2(d)). At the same time, to detect the inflammatory reaction in the cells, we used RT-qPCR and WB to detect the contents of TNF*α* and IL-6 in the cells (Figure 2(d)). We found that exosomes regulated the levels of TNF*α* and IL-6 and reduced the inflammatory response.

**Figure 3:**
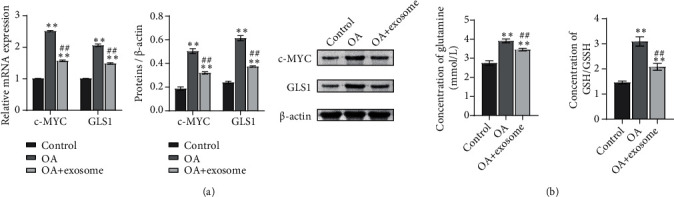
Exosomes affect glutamine metabolism of OA chondrocytes. (a) The contents of c-MYC and GLS1 were detected by biochemical kit. (b) The content of glutamine and GSH/GSSG in cells was detected by biochemical kit. ^∗∗^*P* < 0.01 vs. the control; ^##^*P* < 0.01 vs. OA.

After detecting the changes in cell function, we began to see changes in glutamate metabolism. A biochemical kit was utilized to detect the metabolism of glutamine. We found that the treatment of exosomes reduced the content of c-MYC and GLS1 (Figure 3(a)). In addition, the content of glutamine and GSH/GSSG in the cells was detected. The results showed that glutamine and GSH/GSSG content in the OA group increased while the exosome decreased (Figure 3(b)). These results suggested that exosomes affect glutamine metabolism in OA chondrocytes.

### 3.3. oe-c-MYC Decreased the Therapeutic Effect of Exosomes on OA Chondrocytes and Affected the Function of OA Chondrocytes and Glutamine Metabolism

**Figure 4:**
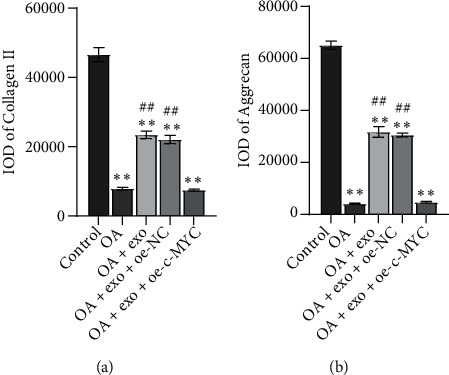
oe-c-MYC decreased the therapeutic effect of exosomes on OA chondrocytes and affected the function of OA chondrocytes. The expressions of collagen II (a) and Aggrecan (b) were detected by IF. ^∗∗^*P* < 0.01 vs. control; ^##^*P* < 0.01 vs. OA.

**Figure 5:**
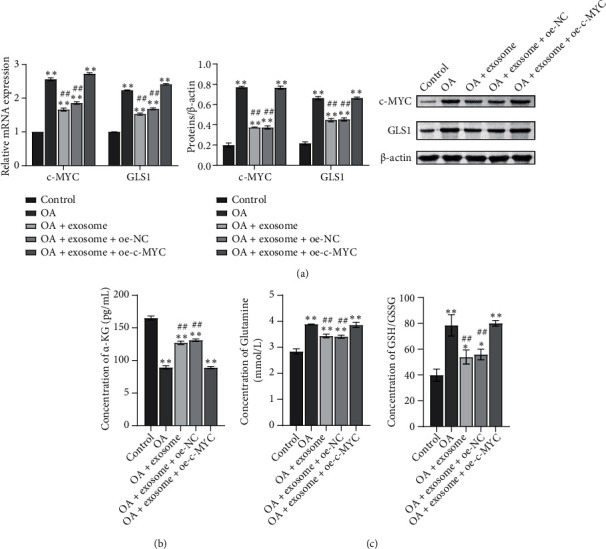
oe-c-MYC decreased the therapeutic effect of exosomes on OA chondrocytes and affected glutamine metabolism. (a) The mRNA and protein contents of c-MYC and GLS1 were detected by RT-qPCR and WB. (b) The content of *α*-KG was detected by ELISA. (c) The contents of glutamine and GSH/GSSG were detected by the biochemical kit. ^∗∗^*P* < 0.01 vs. control; ^##^*P* < 0.01 vs. OA.

A study has shown that exosomes downregulate the expression of c-MYC in breast cancer cells [26], but whether exosomes can affect glutamine metabolism of OA chondrocytes by downregulating c-MYC is unknown. Therefore, we constructed c-MYC overexpression vector (oe-c-MYC) and its control vector (oe-NC). OA+exosome was treated with oe-c-MYC and oe-NC, respectively. The changes of cell function and glutamate metabolism after overexpression of c-MYC were detected. Similarly, to detect cell function changes, we first detected the expressions of collagen II and Aggrecan by IF (Figures 4(a) and 4(b), Figure S2A-B). The results showed that the OA and OA+exosome groups were consistent with those of previous studies, while when exosomes were combined with oe-c-MYC, the positive rates of collagen II and Aggrecan were decreased. The results indicated that oe-c-Myc reduced the therapeutic effect of exosomes on OA chondrocytes and affected the function of cells. Next, to detect the changes in glutamate metabolism in each group, we used RT-qPCR and WB to detect the expression of glutamate metabolism activity-related proteins c-MYC and GLS1 in each group of cells (Figure 5(a)). In the next step, we used ELISA to detect the content of *α*-KG. The results showed that the *α*-KG of the OA group was significantly lower than that of the control group. When the exosome increased the content of *α*-KG, we found that if oe-c-MYC and exosomes were combined with OA chondrocytes, the content of *α*-KG was reduced again (Figure 5(b)). The contents of GLN and GSH/GSSG were detected by the biochemical kit to detect the difference in glutamine uptake. Exosomes decrease the expression of various indicators, but when combined with oe-c-MYC, their expression increased, hindering the therapeutic effect of exosomes (Figure 5(c)).

### 3.4. Exosomes Alleviate Joint Injury in OA Rats

**Figure 6:**
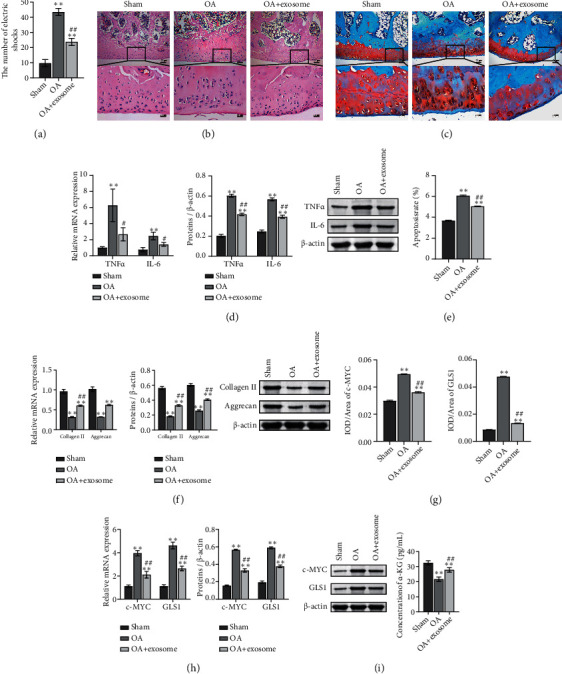
Exosomes alleviate joint injury in OA rats. (a) The movement of mice on the treadmill. When all mice ran 37.69 meters, the number of electric shocks received by the mice was counted. (b) The cartilage was stained with HE. Scale bar = 100 *μ*m and 25 *μ*m. (c) The cartilage was stained with safranin solid green staining. Scale bar = 100 *μ*m and 25 *μ*m. (d) mRNA and protein expressions were detected by RT-qPCR and WB to detect TNF*α* and IL-6. (e) TUNEL assay was used to detect apoptosis. Scale bar = 25 *μ*m. (f) mRNA and protein expressions of collagen II and Aggrecan were detected by RT-qPCR and WB. (g) The expression of c-MYC and GLS1 was detected by IHC. Scale bar = 100 *μ*m and 25 *μ*m. (h) mRNA and protein expressions of c-MYC and GLS1 were detected by RT-qPCR and WB. (i) *α*-KG content was detected by ELISA. ^∗∗^*P* < 0.01 vs. sham. ^##^*P* < 0.01 vs. OA.

The joint injury model of rats was established, and exosomes were injected into both hind limbs' joint cavities to treat OA injury. The three groups were the sham, OA, and OA+exosome groups. Firstly, the motor ability of rats in each group was detected. The results showed that the number of electric shocks in the exosome treatment group was lower than that in the OA group when the movement distance was the same (Figure 6(a)). The knee tissues of rats stained with HE and safranin solid green staining. HE results showed that the joint structure of the sham group was complete, and the chondrocytes were evenly distributed. In the model group, the number of chondrocytes decreased, and the distribution was uneven. After treatment with exosomes, the articular cartilage surface was more complete than that of the model group. The number of cells was distributed evenly, and some had cell clusters (Figure 6(b)). The results of safranin solid green staining showed that the cartilage in the control group and the OA+exosome group was arranged orderly, and the surface structure of cartilage was complete. In the OA group, there were obvious cracks on the surface of the specimens and the arrangement of chondrocytes was disordered (Figure 6(c)). RT-qPCR and WB detected the expression of TNF*α* and IL-6 in tissues. The results showed that exosomes had a significant effect on OA inflammation (Figure 6(d)). The TUNEL kit was used to stain the tissue, and we found that apoptosis in the OA group increased, and exosomes reversed this increase (Figure 6(e), Figure S3A). Furthermore, we detected the levels of collagen II and Aggrecan in tissues by RT-qPCR and WB. The results showed that the treatment of exosomes reversed the decrease of the expression levels of collagen II and Aggrecan by OA (Figure 6(f)). Finally, in order to detect glutamine metabolism-related proteins c-MYC and GLS1, we carried out IHC staining, RT-qPCR detection, and WB detection on the tissues. The results showed that compared with the sham group, c-MYC and GLS1 increased in the OA group, while exosome decreased this increase (Figures 6(g) and 6(h), Figure S3B). The expression of *α*-KG in the OA group was lower than that in the sham group, while exosome increased the content of *α*-KG (Figure 6(i)). These results suggested that exosomes have a positive effect on OA and can regulate glutamine metabolism in the bone and joint.

## 4. Discussion

OA is a global disease. The limitations of current research on OA treatment have resulted in no reliable drugs to cure OA. Exosomes have been a hot research topic in recent years and have therapeutic effects on many diseases. We found that exosomes can affect the metabolism of glutamine. Inflammation is related to the progression of OA, and anti-inflammatory drugs can prevent the development of OA [27]. For example, quercetin [28] and ABT263 (navitoclax) [29] alleviate OA by inhibiting inflammation. Our research first showed that the occurrence of OA triggered inflammation, and the content of inflammatory factors TNF*α* and IL-6 increased in the OA model. The exosomes secreted by MSC have a therapeutic effect on OA and can reduce the levels of TNF*α* and IL-6 in OA. Therefore, in the treatment of OA, exosomes are also equivalent to a new type of anti-inflammatory drug. Exosomes secreted by MSC also have a similar effect in the TMJ-OA treatment, suggesting that exosomes do have anti-inflammatory effects [30].

Apoptosis is positively correlated with the severity of cartilage destruction and matrix depletion [31]. Apoptosis is programmed cell death and an essential life process [32]. But inappropriate apoptosis is a factor in many human diseases, including neurodegenerative diseases, inflammatory diseases, and cancer [32]. Bao et al. pointed out that rapamycin could improve OA by inhibiting rat chondrocytes from IL-18-induced apoptosis [33]. Wang et al. suggested that propolis could inhibit OA by inhibiting mouse chondrocytes from IL-1*β*-induced apoptosis [34]. These results showed that we could improve OA by inhibiting cell apoptosis. Our results confirmed this. We detected the apoptosis of chondrocytes in each group of rats. The results showed that the apoptosis of chondrocytes in OA rats increased, while the exosome treatment group could significantly downregulate the apoptosis of chondrocytes.

Glutamic acid metabolism-related proteins c-MYC and GLS1 were used to characterize the activities of glutamic acid metabolism-related enzymes [35]. A study has shown that Gankyrin can upregulate c-MYC by activating *β*-catenin signal transduction to drive glycolysis and glutamine breakdown [35]. Another study has also shown that the dysregulated XLOC_006390/c-MYC axis increases glutamate metabolism and promotes the progression of pancreatic cancer to a higher stage [36]. In our study, the expression of c-MYC and GLS1 increased in OA, but decreased after treatment with exosomes. It indicated that exosomes affect glutamate metabolism. In addition, when we treated OA chondrocytes with oe-c-MYC and exosomes, the metabolism of glutamate in the cells increased, while the expression of collagen II and Aggrecan decreased, which promoted the progress of OA. Our study showed that oe-c-MYC hindered the therapeutic effect of exosome on OA. Although our study found that MSC-secreted exosomes have a positive therapeutic effect on OA, they still could not cure OA. In the future, we will try to use exosomes in clinical trials of OA and further confirm the therapeutic effect of exosomes on OA. Moreover, we hope that through clinical trials; we can further show that exosomes can reduce the expression of c-MYC to reduce glutamate metabolism, thereby alleviating OA.

## 5. Conclusion

In this study, the effect of MSC-exosomes on chondrocyte function and glutamine metabolism was systematically studied. At the same time, exosomes can affect arthritis chondrocyte function and glutamate metabolism by downregulating c-MYC. It provided a preliminary basis for the subsequent clinical treatment of OA by exosomes.

## Data Availability

All the data is available for publication.

## Supplementary Materials

Supplementary 1 Figure S1: exosomes affect cell function of OA chondrocytes. (A) apoptosis was identified by flow cytometry. (B) Collagen II and Aggrecan of chondrocytes were detected by IF. Scale bar = 25 *μ*m.

Supplementary 2 Figure S2: oe-c-MYC decreased the therapeutic effect of exosomes on OA chondrocytes and affected the function of OA chondrocytes. The expressions of collagen II (A) and Aggrecan (B) were detected by IF. Scale bar = 25 *μ*m.

Supplementary 3 Figure S3: exosomes alleviate joint injury in OA rats. (A) TUNEL assay was used to detect apoptosis. Scale bar = 25 *μ*m. (B) The expression of c-MYC and GLS1 was detected by IHC. Scale bar = 100 *μ*m and 25 *μ*m.
