# Correction to “Mesenchymal Stem Cell-Derived Exosomes Modulate Chondrocyte Glutamine Metabolism to Alleviate Osteoarthritis Progression”

**DOI:** 10.1155/mi/9790328

**Published:** 2025-10-09

K. Jiang, T. Jiang, Y. Chen, and X. Mao, “Mesenchymal Stem Cell-Derived Exosomes Modulate Chondrocyte Glutamine Metabolism to Alleviate Osteoarthritis Progression,” *Mediators of Inflammation* 2021 (2021): 2979124, https://doi.org/10.1155/2021/2979124.

In the article, there are errors in the Abstract, the caption of Figure 6a, and Section 3.1. Specifically:

1. In the Abstract and the legend of Figure 6a, “mice” should be replaced with “rat.”
2. In Section 3.1, “PKH67” should be replaced with “PKH26.”
3. In Section 3.1, “spinal cord” should be replaced with “bone marrow.”

We apologize for these errors.

