# Supplementary figures and images for: Mesenchymal Stem Cell-Derived Exosomes Modulate Chondrocyte Glutamine Metabolism to Alleviate Osteoarthritis Progression
Source: Mediators Inflamm. 2021 Dec 27;2021:2979124. doi: 10.1155/2021/2979124 (PMC8724850; doi:10.1155/2021/2979124)

**A**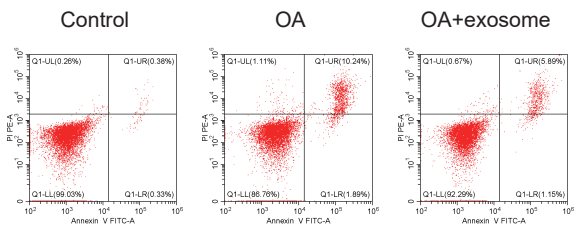**B**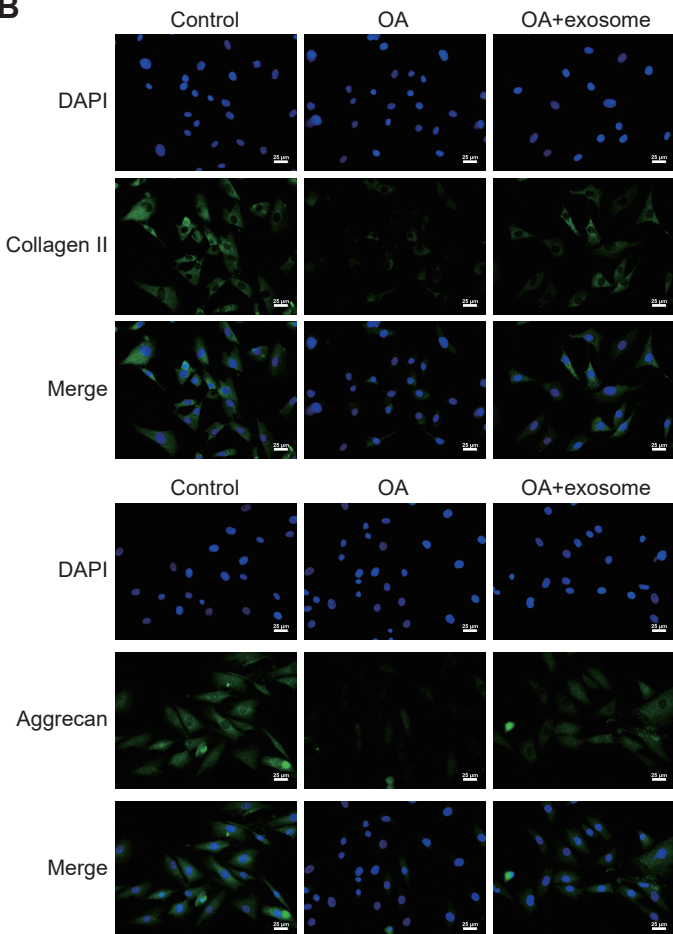

Supplement: Supplementary 1 — Figure S1: exosomes affect cell function of OA chondrocytes. (A) apoptosis was identified by flow cytometry. (B) Collagen II and Aggrecan of chondrocytes were detected by IF. Scale bar = 25 μm. [file 2979124.f1.pdf]

**A**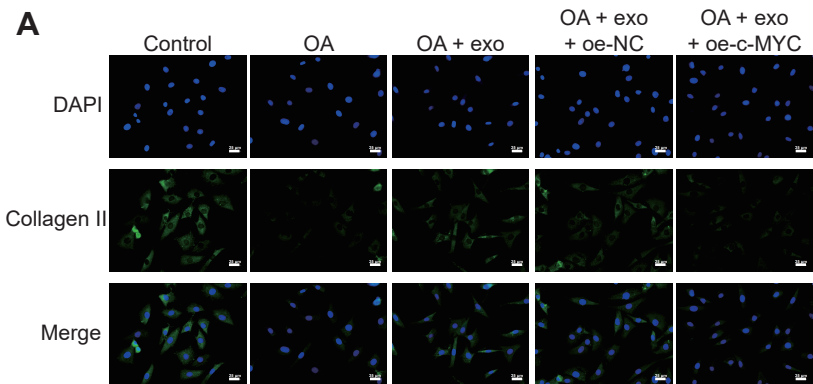**B**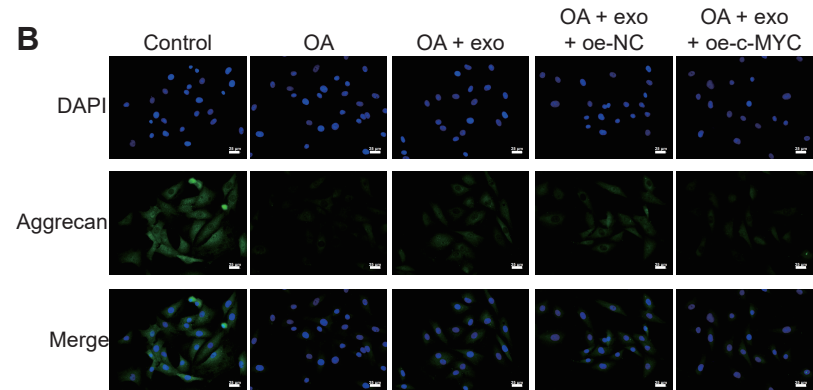

Supplement: Supplementary 2 — Figure S2: oe-c-MYC decreased the therapeutic effect of exosomes on OA chondrocytes and affected the function of OA chondrocytes. The expressions of collagen II (A) and Aggrecan (B) were detected by IF. Scale bar = 25 μm. [file 2979124.f2.pdf]

**A**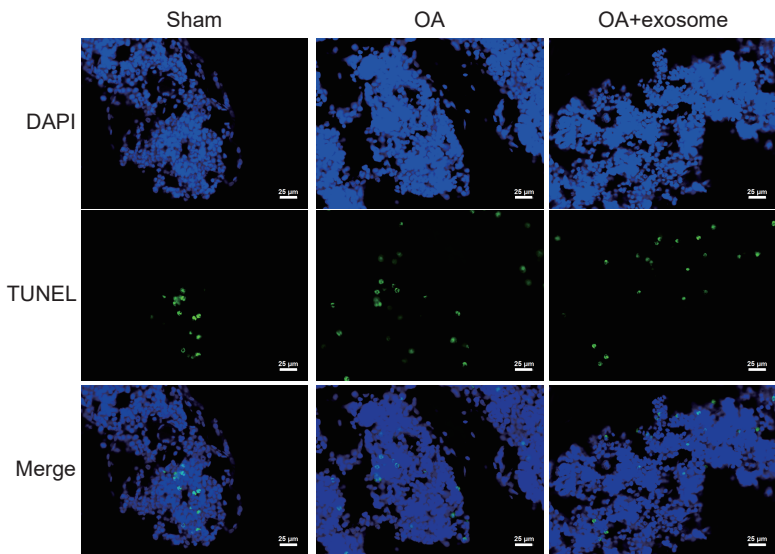**B**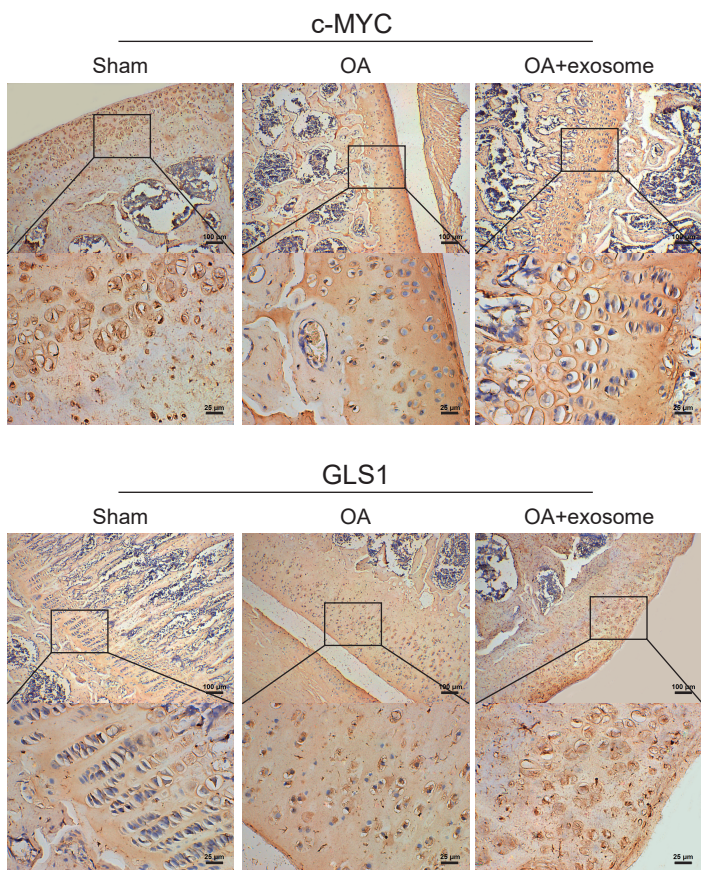

Supplement: Supplementary 3 — Figure S3: exosomes alleviate joint injury in OA rats. (A) TUNEL assay was used to detect apoptosis. Scale bar = 25 μm. (B) The expression of c-MYC and GLS1 was detected by IHC. Scale bar = 100 μm and 25 μm. [file 2979124.f3.pdf]
